# Exogenous Proline Maintains Cell Wall Structure and Membrane Integrity in Rice Seedlings Under Cr(VI) Stress Associated with Regulation of Proline-Rich Proteins

**DOI:** 10.3390/ijms27156669

**Published:** 2026-07-26

**Authors:** Cai-Mei Wang, Xue-Qian Wang, Ben-Tao Yao, Qing Zhang, Yu-Juan Lin, Yan-Peng Liang

**Affiliations:** 1 Guangxi Key Laboratory of Environmental Pollution Control Theory and Technology, Guilin University of Technology, Guilin 541006, China; 2 University Engineering Research Center of Watershed Protection and Green Development, Guangxi, Guilin University of Technology, Guilin 541006, China; 3 Collaborative Innovation Center for Water Pollution Control and Water Safety in Karst Area, Guilin University of Technology, Guilin 541006, China

**Keywords:** heavy metal stress, proline-rich proteins, exogenous proline, cell wall structure

## Abstract

Cr(VI) pollution severely damages plant cell wall structure and membrane integrity. Proline-rich proteins (PRPs), key cell wall structural components, rely on proline as their biosynthetic precursor. Pro accumulation has been shown to positively correlate with PRP abundance, suggesting a direct biochemical link between exogenous Pro [Pro_(exo)_] application and PRP-mediated cell wall function. However, how Pro_(exo)_ regulates PRP expression to maintain cell wall structural integrity and membrane stability in rice seedlings under Cr(VI) stress remains unclear. In this study, the subcellular distribution of Cr, electrolyte leakage (EL), cell wall thickness, and the expression of PRP genes in rice seedlings were evaluated. The results revealed that Cr(VI) stress induced significant increases in EL and changes in cell wall thickness in rice seedling cells (*p* < 0.05). The application of Pro_(exo)_ significantly mitigated EL and increased cell wall thickness (*p* < 0.05). Furthermore, Pro_(exo)_ markedly increased the sequestration of Cr within the seedling root cell walls while reducing its distribution in the cytoplasm and organelles of seedling shoot cells (*p* < 0.05). Phylogenetic analysis of 48 OsPRP genes, on the basis of their homology with seven functionally characterized PRPs from other species, revealed 12 candidate genes in rice potentially involved in the regulation of cell membrane stability, cell wall assembly, thickening, and integrity. Subsequent qRT-PCR analysis and gene expression variation factor calculation revealed that Pro_(exo)_ significantly promoted the expression of *OsPRP1.1*, *OsPRP3*, *OsRePRP2.1*, *OsHyPRP18*, and *OsHyPRP27* in roots and that of *OsHyPRP27* in the shoots of rice seedlings under Cr(VI) stress. These findings indicate that Pro_(exo)_ enhances Cr compartmentalization within the root cell wall and improves membrane stability, thereby contributing to Cr(VI) tolerance in plants through the modulation of these key PRP genes. Our findings provide novel insights into the mechanism by which Pro_(exo)_ regulates PRP expression to affect plant cellular structural stability and heavy metal tolerance.

## 1. Introduction

Chromium (Cr) is a nonessential element for both plants and animals and is potentially toxic. As one of the most common heavy metal (HM) pollutants, Cr is ranked seventh among the top 20 hazardous substances according to the Agency for Toxic Substances and Disease Registry and is receiving considerable scientific and regulatory attention [1]. In the environment, Cr exists primarily in two stable oxidation states, trivalent chromium (Cr(III)) and hexavalent chromium (Cr(VI)), with the toxicity of Cr(VI) being approximately 100 times greater than that of Cr(III). Anthropogenic activities such as mining, metallurgy, electroplating, and textile dyeing wastewater are major sources of Cr pollution [2,3]. Elevated concentrations of Cr in the environment can lead to decreased seed germination rates [4], impaired photosynthetic mechanisms [5], disrupted nutrient uptake and homeostasis [6], increased production of reactive oxygen species (ROS) [7], dysregulation of enzymatic activity [8], and alterations in membrane and cell wall structural stability.

Rice (*Oryza sativa* L.) is among the three major food crops worldwide. Heavy metals (HMs) can severely affect the growth, development, yield, and quality of rice, and ultimately accumulate in the human body through the food chain, causing irreversible harm to human health [9]. When humans are exposed to Cr(VI) through contaminated drinking water or agricultural products, symptoms such as angular stomatitis, diarrhea, abdominal pain, dyspepsia, and vomiting may occur [10,11], potentially leading to malignant intestinal tumors [12] or even death.

The cell wall serves as the first line of defense in plants against HM stress. It is composed mainly of cellulose, hemicellulose, lignin, pectin, and structural proteins [13]. The plant cell wall responds to HM stress through two primary mechanisms: (1) as a signaling medium—cell wall integrity sensors including receptor-like protein kinases (RLKs), rapidly perceive HM-induced structural changes and initiate downstream regulatory responses. For example, the L-type lectin RLK, *OsCORK1*, remodels rice cell wall components under copper (Cu) stress [14] and CrRLK1L supports root growth under Cu, Cd, and Ni metal ion stress [15]; (2) as a “reservoir” for HMs—polysaccharide components such as cellulose, hemicellulose, and pectin, these compounds have functional groups, including hydroxyl (-OH), carboxyl (-COOH), aldehyde (-CHO) and amino (-NH_2_) groups, which serve as binding sites for heavy metal ions, enabling the cell wall to adsorb, precipitate, and chelate HMs and subsequently enter the protoplast [16,17,18]. Among these functional groups, -COOH and -OH play dominant roles, significantly enhancing the ability of the cell wall to immobilize and alleviating the phytoxicity of HMs.

Proline-rich proteins (PRPs) are a group of cell wall proteins belonging to the 8-cysteine motif (8CM) domain protein family and are characterized by high contents of Pro and hydroxyproline. They are associated with tolerance to abiotic stresses such as HMs, drought, and low temperature [19,20]. PRPs are classified into three categories on the basis of their Pro arrangement: (1) PRP, which possesses an N-terminal signal peptide and a proline-rich repeat sequence (PPVXK) [21]; (2) HyPRP, in which the proline-rich repeats lie upstream of the 8CM domain and an atypical proline-rich repeat region occurs at the N-terminus [21]; and (3) RePRP, which contains highly repetitive PX1PX2 motifs in which multiple hydroxyproline residues are extensively glycosylated [22]. Under HM stress, PRP gene expression is activated to alleviate HM toxicity [23]. PRPs act synergistically with cell wall polysaccharides to maintain structural integrity [24,25], and engineering of PRP genes enhances plant stress responsiveness. For instance, CRISPR/Cas9-mediated editing of *SlHyPRP1* generated a salt-tolerant tomato mutant [26], and overexpression of walnut *JsPRP1* in tobacco (*Nicotiana tabacum* L.) alleviated drought and Cd stress [27]. Critically, Pro serves as the key biosynthetic precursor for PRPs: Pro deficiency directly impairs cell wall protein biosynthesis, and Pro accumulation has been shown to be positively correlated with PRP abundance [28]. Pro_(exo)_ treatment significantly promotes the expression of cell wall-related protein genes [29], thereby providing a direct biochemical rationale for investigating how Pro_(exo)_ regulates PRP-mediated cell wall function under Cr(VI) stress.

Pro is a multifunctional amino acid that functions as an osmotic regulator and antioxidant in plant cells [30], participating in nutrient homeostasis [31], carbon and nitrogen metabolism [32], photosynthesis [33], and cell wall synthesis [34]. Under abiotic stress, Pro stabilizes membrane structures and prevents electrolyte leakage (EL) [35], while scavenging ROS and alleviating membrane lipid peroxidation [36]. Pro_(exo)_ application has been widely demonstrated to maintain cell membrane integrity and support cell wall synthesis under HM stress [34,37,38,39]. Our previous work further revealed that Pro_(exo)_ enhances cell wall component synthesis and HM sequestration in rice seedlings under Cr [40,41].

While the roles of Pro_(exo)_ in promoting HM tolerance through osmotic regulation and antioxidation are relatively well established, the mechanisms by which Pro_(exo)_ regulates PRP expression to maintain cell membrane integrity and cell wall structural stability under Cr(VI) stress remain unclear. We hypothesize that Pro_(exo)_ may alleviate Cr(VI)-induced growth inhibition by modulating PRP expression, consequently altering cell wall synthesis and cell membrane permeability. To investigate this, we (1) analyzed the subcellular distribution of Cr, EL and cell wall thickness in rice seedlings; (2) quantified the expression levels of key PRP genes and gene expression variation factors (*GEVFs*); and (3) constructed a phylogenetic tree of PRP family genes. This study aims to elucidate the mechanisms by which Pro-mediated PRP regulation contributes to cellular structural stability in rice seedlings under Cr(VI) stress.

## 2. Results

### 2.1. Subcellular Accumulation of Total Cr in Rice Seedling Tissues

**Figure 1:**
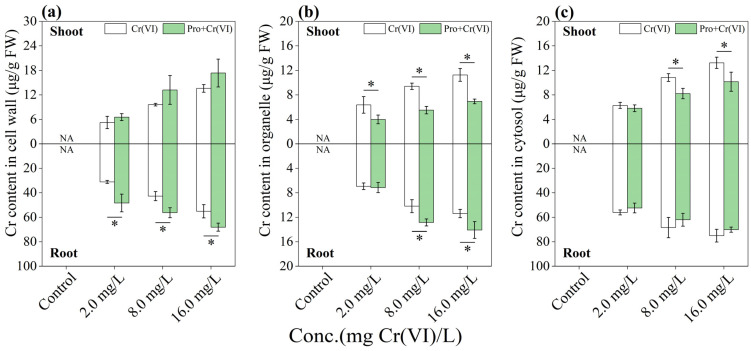
Accumulation of Cr in the cell wall (**a**), organelles (**b**), and cytoplasm (**c**) of subcellular components in rice seedlings under “Cr(VI)” and “Pro + Cr(VI)” treatments. The values represent the mean ± standard deviation (SD) of four independent biological replicates, and asterisks indicate significant differences between treatment groups (*p* < 0.05). NA indicates that Cr was not detected in control samples.

To determine how Pro_(exo)_ affects the subcellular fate of Cr in rice seedlings, we analyzed the distribution of Cr across the cell wall, organelle, and cytoplasmic fractions of roots and shoots. Under both the “Cr(VI)” and “Pro + Cr(VI)” treatments, the Cr content in all three fractions increased with increasing Cr(VI) concentration (Figure 1).

Under the “Cr(VI)” treatment, the distribution proportions of Cr accumulation in the root subcellular fractions (cell wall, organelles, cytoplasm) of the rice seedlings under 2.0, 8.0, and 16.0 mg Cr(VI)/L stress were (33.09%, 7.37%, 59.54%), (35.23%, 8.40%, 56.37%), and (38.93%, 8.05%, 53.02%), respectively. These findings indicate that the order of Cr accumulation in the roots of the rice seedlings was cytoplasm > cell wall > organelles (Figure 1). For the shoot subcellular fractions under the same stress concentrations, the distributions were (29.35%, 35.55%, 35.10%), (32.19%, 31.54%, 36.27%), and (35.68%, 29.56%, 34.76%), respectively. These findings suggest that there was no significant difference in Cr accumulation among the three shoot subcellular fractions (Figure 1).

Under the “Pro + Cr(VI)” treatment, the proportions of Cr in the subcellular components (cell wall, organelles, cytoplasm) of the roots of the rice seedlings under 2.0, 8.0, and 16.0 mg Cr(VI)/L stress were (44.78%, 6.63%, 48.59%), (42.90%, 9.79%, 47.31%), and (44.71%, 9.25%, 46.04%), respectively. The distribution of Cr in the seedling roots decreased in the order of cell wall > cytoplasm > organelles (Figure 1). In the shoots, under the same treatment conditions, the distribution ratios were (40.03%, 24.46%, 35.51%), (49.03%, 20.47%, 30.51%), and (50.41%, 20.12%, 29.47%), respectively. In other words the accumulation of Cr in seedlings shoots followed cell wall > cytoplasm > organelles (Figure 1).

Compared with the “Cr(VI)” treatment, Pro_(exo)_ treatment significantly promoted Cr accumulation in the root cell walls and organelles of the rice seedlings, while reducing Cr accumulation in the shoot organelles and cytoplasm (Figure 1). Specifically, with Pro_(exo)_ pretreatment, Cr accumulation in the root cell walls increased by 55.12%, 31.67%, and 23.62% under 2.0, 8.0, and 16.0 mg Cr(VI)/L stress, respectively. The accumulation of Cr in the root organelles increased by 26.03% and 23.62% under 8.0 and 16.0 mg Cr(VI)/L stress, respectively. However, no significant difference in Cr accumulation was detected in the root cytoplasm across all three treatment concentrations. In the shoots, Cr accumulation in the organelles decreased by 37.09%, 41.41%, and 38.45% under the respective Cr(VI) stress levels, whereas Cr accumulation in the shoot cytoplasm decreased by 24.08% and 23.34% under 8.0 and 16.0 mg Cr(VI)/L stress, respectively. Conversely, no significant difference in Cr accumulation was detected in the shoot cell walls across all concentrations.

These findings suggest that Pro_(exo)_ redistributes Cr across subcellular compartments in a tissue-specific manner, increasing Cr retention within root cell walls while reducing Cr accumulation in the organelles and cytoplasm of shoots, thereby restricting Cr translocation to aerial tissues.

### 2.2. Effects of Pro_(exo)_ on Electrolyte Leakage

**Figure 2:**
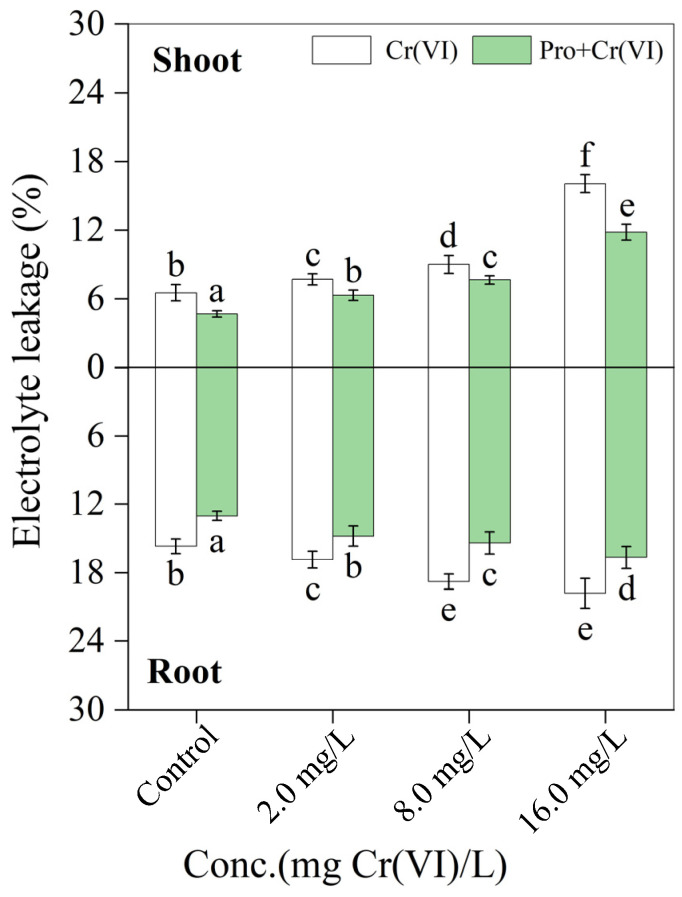
Changes in the EL of rice seedlings under “Cr(VI)”and “Pro + Cr(VI)” treatments. The values represent the mean ± standard deviation (SD) of four independent biological replicates. Different letters indicate significant differences (*p* < 0.05).

To assess whether Pro_(exo)_ preserves cell membrane integrity under Cr(VI) stress, EL was measured as an indicator of membrane damage. Under both the “Cr(VI)” treatment and the “Pro + Cr(VI)” treatment, the EL (%) in the roots and shoots of the rice seedlings increased with increasing Cr(VI) exposure concentration (Figure 2). Compared to Control 1, the “Cr(VI)” treatment resulted in EL increases of 7.63%, 19.71%, and 26.33% in the roots, and 18.10%, 38.11%, and 145.82% in the shoots at 2.0, 8.0, and 16.0 mg Cr(VI)/L, respectively. In the “Pro + Cr(VI)” treatment (compared with that in Control 2), the EL increased by 13.58%, 18.15%, and 27.82% in the roots and by 33.43%, 62.23%, and 150.28% in the shoots at the corresponding Cr(VI) concentrations.

Compared with the “Cr(VI)” treatment, Pro_(exo)_ significantly reduced the EL in both the roots and the shoots of the rice seedlings (*p* < 0.05) (Figure 2). Specifically, with Pro_(exo)_ pretreatment, the EL in roots decreased by 16.84%, 12.24%, 17.92%, and 15.85% at 0.0, 2.0, 8.0, and 16.0 mg Cr(VI)/L, respectively. Moreover, the EL in the seedling shoots decreased by 27.67%, 18.29%, 15.04%, and 26.36% at the corresponding Cr(VI) concentrations.

These results indicate that Pro_(exo)_ alleviates Cr(VI)-induced cell membrane damage in rice seedlings.

### 2.3. Effects of Pro_(exo)_ on the Cell Wall Structure of Rice Seedlings

**Figure 3:**
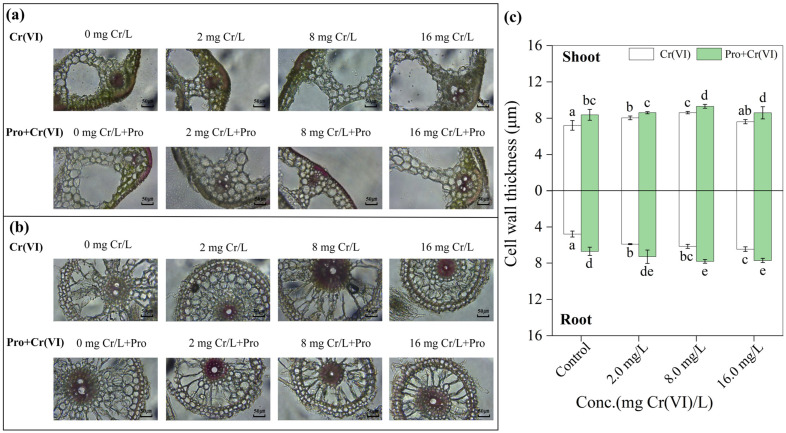
Microscopic imaging (400×) of the cell walls in shoot (**a**) and root (**b**) tissues of rice seedlings treated with “Cr(VI)”and “Pro + Cr(VI)” and (**c**) the determination results of the cell wall thickness of the rice seedlings. The values represent the mean ± standard deviation (SD) of four independent biological replicates. Different letters indicate significant differences (*p* < 0.05).

To investigate whether Pro_(exo)_ influences cell wall structural remodeling under Cr(VI) stress, cell wall thickness was measured in root and shoot tissues. Under the “Cr(VI)” treatment, the cell wall thickness of the roots of the rice seedlings increased significantly (*p* < 0.05) with increasing Cr(VI) concentrations (Figure 3). Compared with that in Control 1, the root cell wall thickness increased by 23.46%, 28.48%, and 34.99% at 2.0, 8.0, and 16.0 mg Cr(VI)/L, respectively. In the shoots, the cell wall thickness increased significantly by 11.53% and 19.72% at 2.0 and 8.0 mg Cr(VI)/L, respectively, but no significant difference was observed at 16.0 mg Cr(VI)/L.

Under the “Pro + Cr(VI)” treatment, the cell wall thickness of the roots of the rice seedlings increased significantly with increasing Cr(VI) exposure. Compared with that of Control 2, the thickness of the root cell wall increased by 8.81%, 16.52%, and 14.94% at 2.0, 8.0, and 16.0 mg Cr(VI)/L, respectively. In the shoots, a significant increase in cell wall thickness (*p* < 0.05) was observed only at the 8.0 mg Cr(VI)/L concentration, with no significant differences (*p* > 0.05) at the other concentrations (Figure 3).

Compared with the “Cr(VI)” treatment group, Pro_(exo)_ significantly (*p* < 0.05) increased the cell wall thickness in both the roots and the shoots of the rice seedlings under 0, 2.0, 8.0, and 16.0 mg Cr(VI)/L stress conditions (Figure 3). Specifically, the root cell wall thickness increased by 40.02%, 23.41%, 26.99%, and 19.22%, whereas the shoot cell wall thickness increased by 16.31%, 7.21%, 7.99%, and 12.75%, respectively, at the different Cr(VI) concentrations.

Pro_(exo)_ increased the cell wall thickness of rice seedlings under Cr (VI) stress, combined with changes in the distribution of Cr in subcellular components, as described in Section 2.1, suggesting a coordinated response between cell wall thickening and Cr immobilization.

### 2.4. Phylogenetic Tree Analysis of PRPs Involved in Cell Wall Structure Regulation in Rice Seedlings

**Figure 4:**
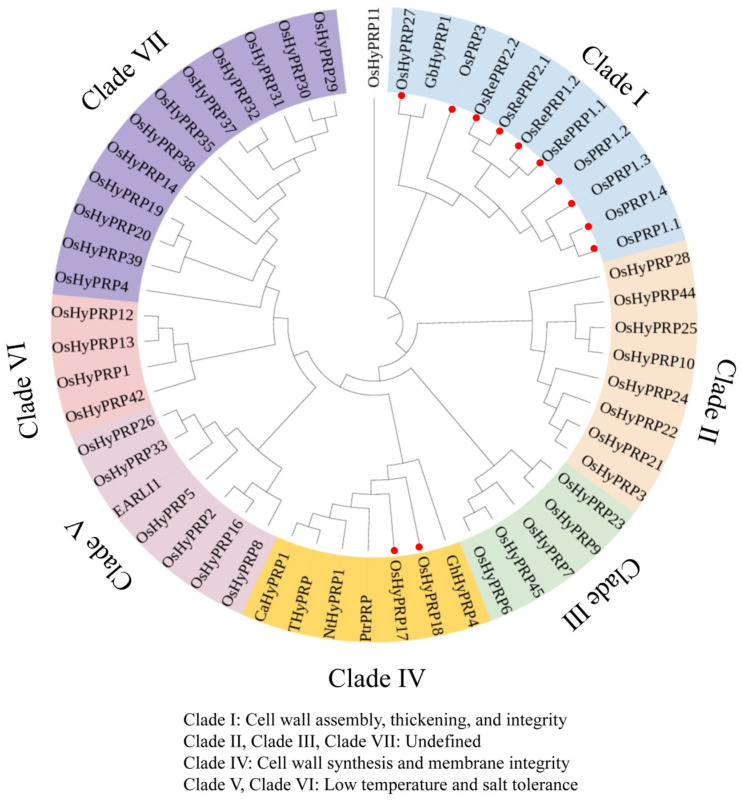
Phylogenetic tree of PRP family genes in rice and other plants. The 12 genes marked with red dots are candidate genes involved in plasma membrane stability and cell wall assembly, thickening, and integrity regulation in rice.

To identify PRP candidate genes potentially involved in the Pro-mediated response to Cr(VI) stress, we constructed a phylogenetic tree of the 48 OsPRP genes alongside seven functionally characterized PRPs from other plant species (Figure 4, Table 1). In Clade I, ten genes (*OsHyPRP27*, *OsPRP3*, *OsRePRP2.2*, *OsRePRP2.1*, *OsRePRP1.2*, *OsRePRP1.1*, *OsPRP1.2*, *OsPRP1.3*, *OsPRP1.4*, and *OsPRP1.1*) showed high homology with *GbHyPRP1*, suggesting their potential involvement in regulating cell wall assembly, thickening, and integrity. In Clade IV, two genes (*OsHyPRP17* and *OsHyPRP18*) exhibited high homology with *CaHyPRP1*, *THyPRP*, *NtHyPRP1*, *PtrPRP*, and *GhHyPRP4,* indicating possible functions in cell wall synthesis and membrane integrity maintenance. Genes within Clades V and VI were highly homologous to *Arabidopsis EARLI* genes, which are associated with tolerance to low temperature and salt stress.

This analysis identified a set of candidate genes for subsequent investigation of the association between Pro_(exo)_ treatment and changes in PRP gene expression under Cr(VI) stress.

### 2.5. Effects of Pro_(exo)_ on the Expression of PRP Genes Involved in Cell Wall Structure Regulation Under Cr(VI) Stress in Rice Seedlings

**Figure 5:**
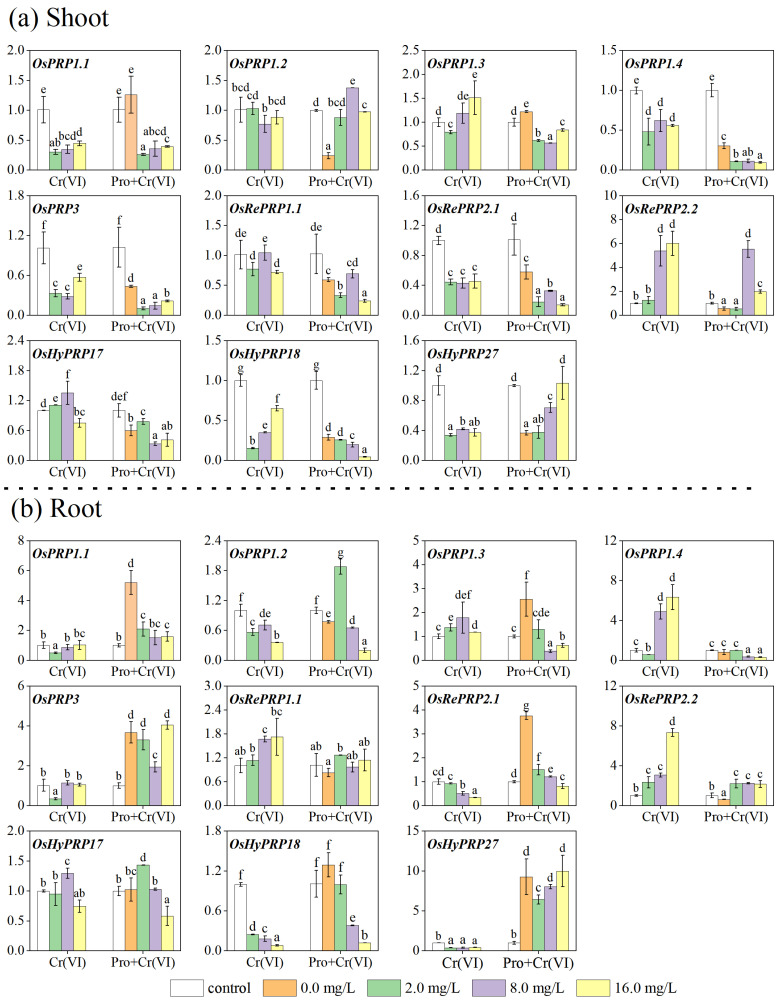
Changes in the relative expression levels of PRP genes involved in cell wall regulation in rice seedling shoots (**a**) and roots (**b**) under “Cr(VI)” and “Pro + Cr(VI)” treatments. The vertical error bars represent the standard deviation. Different lowercase letters indicate significant differences (*p* < 0.05).

To determine which PRP genes are most responsive to Pro_(exo)_ treatment under Cr(VI) stress, we performed qRT-PCR on the 11 candidate genes identified in Section 2.4. The expression levels of these cell wall-regulating PRP genes in the roots and shoots of rice seedlings under “Cr(VI)” and “Pro + Cr(VI)” treatments are shown in Figure 5. Under “Cr(VI)” treatment, in the roots, the expression of *OsPRP1.3* and *OsRePRP2.2* was significantly upregulated after exposure to 2.0, 8.0, and 16.0 mg Cr(VI)/L; that of *OsRePRP1.1* was significantly upregulated after exposure to 8.0 and 16.0 mg Cr(VI)/L; that of OsPRP1.4 was significantly upregulated after exposure to 8.0 and 16.0 mg Cr(VI)/L but significantly downregulated after exposure to 2.0 mg Cr(VI)/L; that of *OsHyPRP17* was significantly upregulated after exposure to 8.0 mg Cr(VI)/L but significantly downregulated after exposure to 16.0 mg Cr(VI)/L; and that of *OsPRP1.2*, *OsHyPRP18*, and *OsHyPRP27* were significantly downregulated after exposure to 2.0, 8.0, and 16.0 mg Cr(VI)/L; that of *OsPRP1.1* and *OsPRP3* was significantly downregulated after exposure to 2.0 mg Cr(VI)/L; and that of *OsRePRP2.1* was significantly downregulated after exposure to 8.0 and 16.0 mg Cr(VI)/L. In the shoots, the expression of *OsRePRP2.2* was significantly upregulated under exposure to 8.0 and 16.0 mg Cr(VI)/L; that of *OsRePRP17* was significantly upregulated under exposure to 2.0 and 8.0 mg Cr(VI)/L but significantly downregulated under exposure to 16.0 mg Cr(VI)/L; that of *OsPRP1.3* was significantly upregulated under exposure to 16.0 mg Cr(VI)/L but significantly downregulated under exposure to 2.0 mg Cr(VI)/L; that of *OsPRP1.1*, *OsPRP1.4*, *OsPRP3*, *OsRePRP2.1*, *OsHyPRP18*, and *OsHyPRP27* was significantly downregulated under exposure to 2.0, 8.0, and 16.0 mg Cr(VI)/L; that of *OsRePRP1.1* was significantly downregulated under exposure to 16.0 mg Cr(VI)/L; and that of *OsPRP1.2* was not significantly different under exposure to 2.0, 8.0, and 16.0 mg Cr(VI)/L.

Under the “Pro + Cr(VI)” treatment, in the roots, the expression of *OsPRP3* and *OsHyPRP27* was significantly upregulated after exposure to 0.0, 2.0, 8.0, and 16.0 mg Cr(VI)/L; that of *OsPRP1.1* was significantly upregulated after exposure to 0.0, 2.0, and 16.0 mg Cr(VI)/L; that of *OsPRP1.2* was significantly upregulated after exposure to 2.0 mg Cr(VI)/L but significantly downregulated after exposure to 0.0, 8.0, and 16.0 mg Cr(VI)/L; that of *OsPRP1.3* was significantly upregulated after exposure to 0.0 mg Cr(VI)/L but sig-nificantly downregulated after exposure to 8.0 and 16.0 mg Cr(VI)/L; that of *OsRePRP2.1* was significantly upregulated after exposure to 0.0, 2.0, and 8.0 mg Cr(VI)/L but significantly downregulated after exposure to 16.0 mg Cr(VI)/L; that of *OsRePRP2.2* was signifi-cantly upregulated after exposure to 2.0, 8.0, and 16.0 mg Cr(VI)/L but significantly down-regulated exposed to 0.0 mg Cr(VI)/L; that of *OsHyPRP17* was significantly upregulated after exposure to 2.0 mg Cr(VI)/L but significantly down-regulated exposed to 16.0 mg Cr(VI)/L; that of *OsPRP1.4* was significantly upregulated after exposure to 8.0 and 16.0 mg Cr(VI)/L; that of *OsHyPRP17* was significantly upregulated after exposure to 16.0 mg Cr(VI)/L; and that of *OsRePRP1.1* showed no significant difference exposed to 0.0, 2.0, 8.0, and 16.0 mg Cr(VI)/L. In the shoots, the expression of *OsPRP1.2* was significantly upregulated after exposure to 8.0 mg Cr(VI)/L but significantly down-regulated exposed to 0.0 mg Cr(VI)/L; that of *OsPRP1.3* was significantly upregulated exposed to 0.0 mg Cr(VI)/L but significantly down-regulated exposed to 2.0, 8.0, and 16.0 mg Cr(VI)/L; that of OsRePRP2.2 was significantly upregulated after exposure to 8.0 and 16.0 mg Cr(VI)/L but significantly downregulated after exposure to 0.0 and 2.0 mg Cr(VI)/L; that of *OsPRP1.4*, *OsPRP3*, *OsRePRP2.1*, *OsHyPRP17*, and *OsHyPRP18* were significantly downregulated after expo-sure to 0.0, 2.0, 8.0, and 16.0 mg Cr(VI)/L; that of *OsRePRP1.1* was significantly downregulated after exposure to 0.0, 2.0, and 16.0 mg Cr(VI)/L; that of *OsPRP1.1* was significantly downregulated after exposure to 2.0, 8.0, and 16.0 mg Cr(VI)/L; and that of *OsHyPRP27* was significantly downregulated after exposure exposed to 0.0, 2.0, and 8.0 mg Cr(VI)/L.

Compared with “Cr(VI)” treatment, Pro_(exo)_ promoted the expression of *OsPRP1.1*, *OsPRP3*, *OsRePRP2.1*, and *OsHyPRP27* in the roots of rice seedlings but suppressed the expression of *OsPRP1.4*, *OsRePRP2.2*, and *OsRePRP1.1*, with no significant changes observed in the expression of *OsPRP1.2*, *OsPRP1.3*, *OsHyPRP17*, and *OsHyPRP18*. In the shoots, Pro_(exo)_ suppressed the expression of *OsPRP1.3*, *OsRePRP2.2*, and *OsHyPRP17*, but caused no significant alterations in the expression of *OsPRP1.1*, *OsPRP1.2*, *OsPRP1.4*, *OsPRP3*, *OsRePRP1.1*, *OsRePRP2.1*, *OsHyPRP18*, and *OsHyPRP27*.

In summary, Pro_(exo)_ primarily promoted the expression of the *OsPRP1.1*, *OsPRP3*, *OsRePRP2.1* and *OsHyPRP27* genes in roots, with distinct tissue-specific expression patterns observed between roots and shoots.

### 2.6. Effect of Pro_(exo)_ on the Gene Expression Variation Factors (GEVFs) of PRP Family Members in Rice Under Cr(VI) Stress

**Figure 6:**
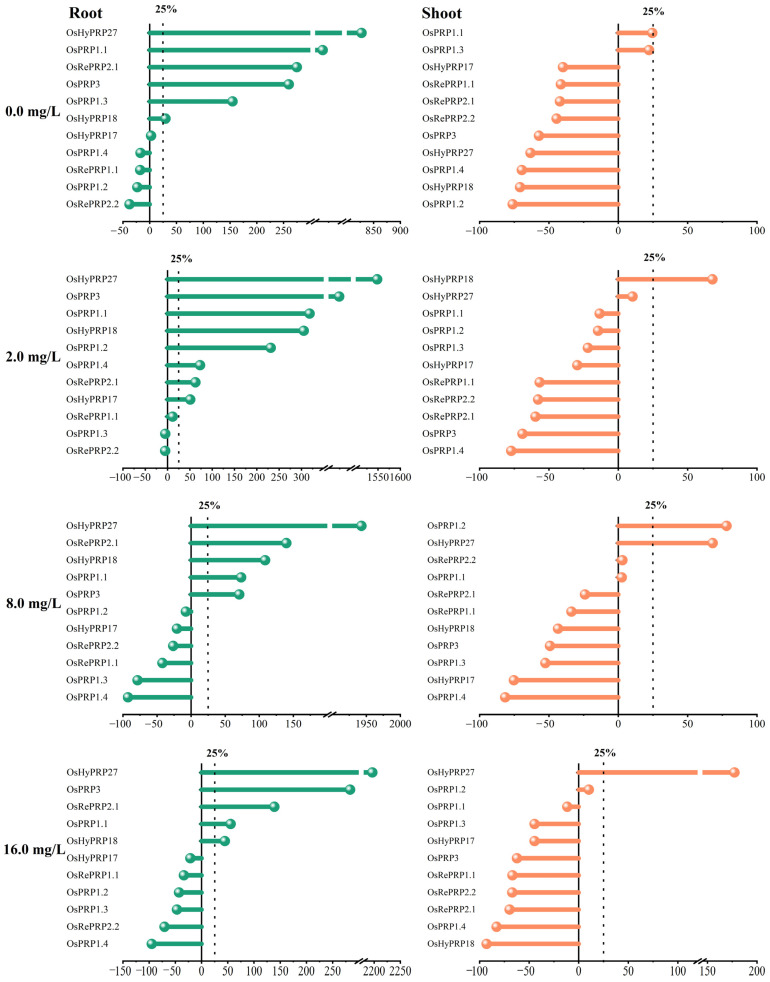
Gene expression variation factors in the expression of PRP genes involved in cell wall regulation in rice seedling tissues under “Cr(VI)” and “Pro + Cr(VI)” treatments.

**Figure 7:**
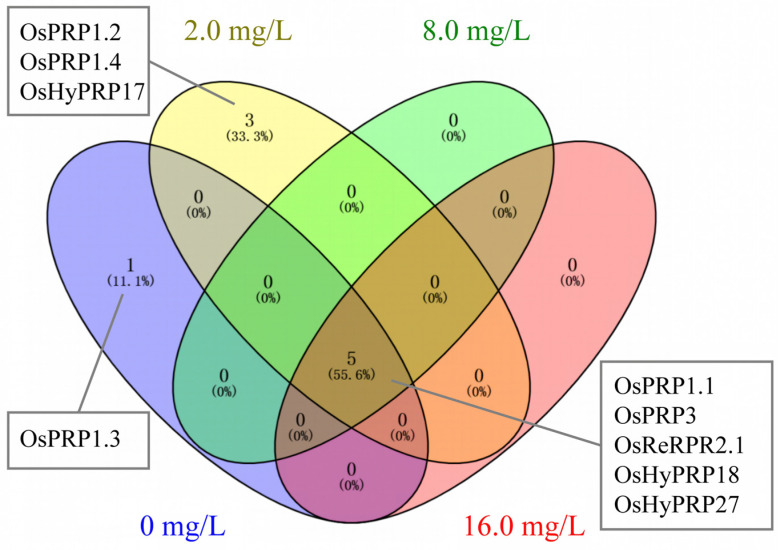
Comparison of PRP genes with GEVFs greater than 25% in the roots of rice seedlings under 0.0, 2.0, 8.0 and 16.0 mg Cr(VI)/L stress.

To identify which PRP genes respond most consistently to Pro_(exo)_ across different Cr(VI) concentrations, *GEVFs* were calculated to quantify the magnitude of Pro-induced expression changes in cell wall-related PRP genes in rice seedlings (Figure 6). The *GEVF* values indicated that a subset of these genes exhibited significantly enhanced responsiveness to Pro pretreatment. Under the “Pro + Cr(VI)” treatment, the rice seedlings exposed to 0.0, 2.0, 8.0, and 16.0 mg Cr (VI)/L showed high *GEVF* values (>25%) for 6 (*OsHyPRP27*, *OsPRP1.1*, *OsRePRP2.1*, *OsPRP3*, *OsPRP1.3*, *OsHyPRP18*), 8 (*OsPRP1.1*, *OsPRP1.2*, *OsPRP1.4*, *OsPRP3*, *OsRePRP2.1*, *OsHyPRP17*, *OsHyPRP18*, *OsHyPRP27*), 5 (*OsPRP1.1*, *OsPRP3*, *OsRePRP2.1*, *OsHyPRP18*, *OsHyPRP27*), and 5 (*OsPRP1.1*, *OsPRP3*, *OsRePRP2.1*, *OsHyPRP18*, *OsHyPRP27*) genes in their roots, respectively. We performed Venn diagram analysis on genes with high *GEVFs* among the different treatment groups, and reported that the expression of *OsPRP1.1*, *OsPRP3*, *OsRePRP2.1*, *OsHyPRP18*, and *OsHyPRP27* in the roots of rice seedlings treated with 2.0, 8.0, and 16.0 mg Cr(VI)/L was significantly promoted by Pro_(exo)_ (Figure 7). In the shoots, exposure to 0.0, 2.0, 8.0, and 16.0 mg Cr(VI)/L resulted in 0, 1 (*OsHyPRP18*), 2 (*OsPRP1.2*, *OsHyPRP27*), and 1 (*OsHyPRP27*) genes showed relatively high *GEVF* values (>25%), respectively. Taken together, the results of the GEVFs analysis revealed that *OsPRP1.1*, *OsPRP3*, *OsRePRP2.1*, *OsHyPRP18*, and *OsHyPRP27* were the core root-expressed PRP genes that were consistently responsive to Pro_(exo)_ across all the Cr(VI) stress concentrations tested, whereas shoot tissues presented markedly fewer and non-overlapping responsive genes, highlighting the tissue-specific nature of the Pro-PRP regulatory response under Cr(VI) stress.

## 3. Discussion

HM contamination is a global concern. As a staple food for more than half of the world’s population [49], rice causes significant yield and quality losses because of Cr stress. Biomass serves as a key indicator for assessing Cr toxicity in plants. Studies have shown that Pro_(exo)_ application significantly alleviates Cr(VI)-induced suppression of the relative growth rate of rice plants [40]. Additionally, Pro enhances plant stress tolerance by acting as an osmoprotectant, metal chelator, and ROS scavenger [50]. Cr(VI) stress triggers excessive ROS accumulation in plants, leading to lipid peroxidation and cellular membrane damage, which subsequently impairs cell elongation and division, thereby hindering plant growth and development [51]. The selective permeability of cell membranes prevents the free movement of extracellular substances. Under HM stress, oxidative stress-induced membrane lipid peroxidation compromises this selective function, increasing membrane permeability and allowing harmful substances to enter the cell [52]. EL serves as an effective indicator for assessing the extent of cell membrane damage under stress conditions. Cr stress-induced membrane damage has been shown to increase EL and reduce membrane stability in cabbage [53]. The application of Pro_(exo)_ not only significantly reduced EL in fenugreek (*Trigonella foenum-graecum*) seedlings under herbicide stress [54], but also increased the activity of antioxidant enzymes (SOD, POD, APX, and CAT) and alleviated heavy metal-induced membrane lipid peroxidation [30]. Our previous study demonstrated that Pro_(exo)_ significantly decreased malondialdehyde accumulation in rice seedlings under Cr(VI) stress [55]. The results of the present study revealed that the “Cr(VI)” treatment significantly increased the EL in rice seedling tissues (Figure 2), whereas the addition of Pro_(exo)_ significantly reduced the EL (*p* < 0.05) (Figure 2). These results are consistent with the antioxidant and osmoregulatory roles of Pro documented in previous studies, suggesting that Pro_(exo)_ may mitigate Cr(VI)-induced membrane lipid peroxidation, thereby alleviating cell membrane damage in rice seedlings.

The distribution of HMs in plant subcellular components is directly related to their toxicity. In general, a large proportion of HMs are sequestered by the plant cell wall. For example, under Cd stress, Cd is distributed primarily in the root cell walls of soybean plants [56]. In pepper plants, the accumulated Cd content in the cell walls of roots, stems, leaves, and fruits is greater than that in organelles and the cytoplasm [57]. Under Cd and Zn stress, the accumulation of HMs in the cell walls of castor bean (*Ricinus communis*) is greater than that in other subcellular fractions [58]. However, the application of exogenous growth regulators can increase the retention of HMs in root cell walls, thereby alleviating the adverse effects of HM stress on plant growth and development. Exogenous auxin (α-naphthaleneacetic acid) stimulates the synthesis of hemicellulose 1, increasing the fixation of Cd in the root cell walls of *Arabidopsis thaliana* [59]. Exogenous melatonin promotes the synthesis of root cell wall components in rice seedlings, enhancing the adsorption of boron (B) by the cell wall [60]. Pro_(exo)_ alters the distribution of Cr in rice seedlings under Cr(VI) stress, with more Cr detected in the root cell walls [61]. Our experimental results revealed distinct differences in the subcellular distribution of Cr in rice seedling tissues between the “Cr(VI)” and “Pro + Cr(VI)” treatments (Figure 1). Under “Cr(VI)” stress, Cr in the root subcellular fractions followed the distribution order of: cytoplasm > cell wall > organelles, with cell wall proportions ranging from 33.09% to 38.93% across the three Cr(VI) concentrations tested. Under the “Pro + Cr(VI)” treatment, the proportion retained in the root cell wall fraction increased substantially to 42.90–44.78%, representing a 5.78–11.69% increase relative to that in the corresponding “Cr(VI)” treatment groups, indicating a significant shift in Cr partitioning toward the cell wall. Conversely, in the shoots, Cr accumulation decreased significantly (*p* < 0.05) in both organelles and the cytoplasm. Our previous study revealed that Pro_(exo)_ promotes Cr accumulation in rice seedling roots (14.86–44.83% ↑) and reduces Cr translocation to shoots (13.6–79.7% ↓) [55]. Taken together, these findings suggest that Pro_(exo)_ enhances Cr compartmentalization within root cell walls, restricts Cr translocation to aerial tissues, and thereby increases the ability of the root system to immobilize Cr under Cr(VI) stress.

It should be noted that while differential centrifugation remains a widely adopted standard for subcellular metal partitioning [62], the homogenization process may induce partial metal redistribution among fractions, and formal purity assessments (e.g., contamination assessment, fraction recovery rates, etc.) were not conducted herein. Nevertheless, because all samples were processed under strictly identical conditions, any potential re-distribution acts uniformly across treatments; therefore, the observed relative shifts in Cr allocation—particularly the pronounced enrichment in the root cell wall fraction with Pro_(exo)_ application (Figure 1a)—reflect biologically meaningful trends rather than absolute compartmental quantitation. In the future, in situ analytical techniques such as synchro-tron XAFS and NanoSIMS will be crucial for the spatial resolution and independent verification of these fractionation-based observations.

Pro serves as an important precursor for the synthesis of cell wall structural proteins, including PRPs. Under abiotic stress, the transport of Pro between various organelles is crucial for plants to maintain stable intracellular Pro levels, protect their cellular structures, and promote cell wall synthesis [63]. The thickening of the plant cell wall induced by abiotic stress is considered an adaptive mechanism [64]. Studies have shown that HM contamination can lead to increased cell wall thickness and expanded cell area in algae [65]. Located at the outermost layer of the cell, the plant cell wall has a composition and structure that are constantly in a dynamic state of change. Adverse stress conditions can alter the components and structure of the cell wall [66], affecting plant stress resistance. For instance, under Cd stress, the pectin, hemicellulose, and cellulose contents in tomatoes increased by 3.41, 2.79, and 1.11 times, respectively, promoting Cd adsorption by the cell wall. This process is facilitated by the activation of key transcription factors that regulate the expression of cell wall biosynthesis genes, driving polysaccharide deposition and cell wall thickening [67], thereby increasing Cd tolerance. The application of Pro_(exo)_ can further alter the content and structure of cell wall components. For example, Pro_(exo)_ promotes the accumulation of pectin and cellulose, improving the rigidity and extensibility of the cell wall and thus alleviating the inhibition of root elongation in plants induced by B deficiency [68,69]. Our previous work has provided systematic biochemical evidence for Pro_(exo)_-induced cell wall remodeling under Cr(VI) stress: Pro_(exo)_ significantly increased the cellulose and lignin contents in rice seedling roots and shoots, with extensin–pectin covalent cross-linking further contributing to cell wall structural reinforcement [29]; in addition, Pro_(exo)_ promoted pectin synthesis and, through regulation of pectin methylesterase activity, increased pectin demethylation, thereby increasing the density of carboxyl (–COOH) binding sites available for Cr chelation within the cell wall [41]. The results of the present study revealed that Pro_(exo)_ significantly increased cell wall thickness in rice seedling tissues (*p* < 0.05) (Figure 3), a structural change that is consistent with the compositional remodeling documented in those previous studies. Notably, this cell wall thickening is concurrent with the substantially increased Cr sequestration in root cell walls demonstrated in Section 2.1, suggesting that Pro_(exo)_ may increase cell wall thickness through polysaccharide remodeling, thereby further enhancing the Cr immobilization capacity of the root cell wall. Under Cr stress, *Cosmos bipinnatus* employs a strategy involving enhanced synthesis of cellulose, hemicellulose, and pectin, along with modifications to the stability of negatively charged groups in the cell wall. This leads to increased Cr accumulation within the cell wall, prevents Cr from entering the cytoplasm, helps maintain the integrity of the cell structure, and ultimately alleviates Cr-induced phytotoxicity [70]. These converging lines of evidence indicate that Pro_(exo)_ increases cell wall thickness and enhances cell wall function through coordinated changes in polysaccharide composition, pectin methylation status, and structural protein expression.

PRPs are widely present in plants and play important roles in the stability of plant cell structure, cell wall assembly during growth and development, and signal transduction within the cell wall [71,72]. Numerous studies have shown that the expression of PRP-related genes is involved in the defense response to external stresses. For example, when soybean (*Glycine max*) is subjected to environmental stimuli, the expression of *GmPRP* rapidly increases, increasing its protein levels to mitigate damage caused by adverse conditions [73]. The expression of PRP genes can defend soybean plants against viral infection [74], increase tobacco tolerance to drought, cold, and osmotic stress as well as methyl viologen-induced oxidative stress [75], and play a role in cell expansion and elongation during early pepper development [76]. Both the overexpression and the underexpression of PRP genes can affect plant physiological traits. For example, overexpression of the cotton gene *GhPRP5* in transgenic *Arabidopsis* resulted in smaller cells and reduced cell growth, leading to plant dwarfing, whereas downregulation of *GhPRP5* promoted fiber development [77]. Studies have shown that PRPs participate in cell wall synthesis and assembly [34]. In poplar, overexpression of the PRP gene *PdPRP* increased stem height and width, promoted the expansion and thickening of the secondary wall, and induced the expression of genes related to the microfibril angle (MFA) and secondary wall biosynthesis [24]. Fungi can induce oxidative cross-linking of PRPs in the cell walls of pea (*Pisum sativum*) or soybean, increasing cell wall reinforcement and damage repair [78]. *OsRePRP* influences cellulose synthesis by regulating sucrose synthase activity, thereby altering the cell wall structure [79]. The overexpression of *OsPRP3* increased the structural integrity and osmotic balance of rice cells under cold stress [80]. Such modifications in these structural proteins of the cell wall are considered among the earliest defense responses of plant cells.

In this study, qRT-PCR and analysis of *GEVFs* were performed to investigate the expression patterns of PRP genes in rice, with the aim of exploring the mechanism by which Pro_(exo)_ is associated with changes in cell wall-related gene expression under Cr(VI) stress. The result of *GEVFs* analysis revealed that *OsPRP1.1*, *OsPRP3*, *OsRePRP2.1*, *OsHyPRP18*, and *OsHyPRP27* were the PRP genes most positively responsive to Pro_(exo)_ in the rice roots, and *OsHyPRP27* was the sole responsive gene in the shoots (Figure 6 and Figure 7). Notably, the expression of *OsHyPRP18* did not significantly differ between the “Cr(VI)” and “Pro + Cr(VI)” treatments in the direct qRT-PCR comparison presented in Section 2.5. However, GEVF analysis, which quantifies the relative magnitude of expression level shifts between two treatment conditions—including cases where both conditions suppress baseline expression but to differing degrees—revealed that *OsHyPRP18* expression in roots under the “Pro + Cr(VI)” treatment was substantially greater than under the same Cr(VI) concentration alone at all concentrations tested. Phylogenetic tree analysis revealed that *OsPRP1.1*, *OsPRP3*, *OsRePRP2.1*, and *OsHyPRP27* cluster with PRPs previously implicated in cell wall assembly and thickening (Clade I), whereas *OsHyPRP18* clusters with PRPs associated with the cell membrane integrity (Clade IV) (Figure 4). These phylogenetic affiliations, combined with *GEVFs*-identified Pro responsiveness, suggest that Pro_(exo)_ may promote cell wall synthesis and structural stability through promoting the expression of *OsPRP1.1*, *OsPRP3*, *OsRePRP2.1*, and *OsHyPRP27* in roots, and may contribute to cell membrane integrity through associated changes in expression of *OsHyPRP18*.

The tissue-specific expression patterns observed in this study also merit discussion. *OsHyPRP27* was markedly upregulated in the roots under the “Pro + Cr(VI)” treatment but showed no significant response in the shoots under the same conditions. This differential regulation may reflect the distinct functional demands of the two tissue types under Cr(VI) stress: root tissues, as the primary sites of Cr uptake, may preferentially activate PRP-mediated cell wall reinforcement to limit Cr entry and restrict its translocation to aerial parts, whereas shoot tissues may employ alternative adaptive strategies. This interpretation is supported by the subcellular distribution data in Section 2.1, where Pro_(exo)_ significantly increased Cr sequestration in root cell walls while reducing Cr accumulation in shoot organelles and the cytoplasm—a spatially differentiated pattern consistent with tissue-specific PRP regulation. Whether this differential expression reflects differences in local Pro accumulation, tissue-specific transcription factor activity, or differential promoter responsiveness remains to be investigated.

Previous studies have indicated that PRP genes can regulate the interaction between the cell wall and the plasma membrane. *NtProRP1* likely regulates pollen germination and pollen tube growth by promoting the normal development of the tobacco cell wall and plays a key role in the osmotic stress response during pollen tube growth [81]. The EARLI1-type *HyPRP* possesses a dual-domain structure: the proline-rich domain (PRD) can interact with the cell wall, whereas the C-terminal eight-cysteine motif can interact with the plasma membrane, collectively protecting the cell during freezing stress [82]. *HyPRPs* are analogous to nonspecific lipid transfer proteins (LTPs) and may be involved in membrane binding or antioxidant responses [83,84,85]. Therefore, elucidating the function of the C-terminal domain of *HyPRPs* (e.g., lipid binding) to elucidate the mechanisms underlying cell wall-plasma membrane interactions represents a key direction for our future research.

The present study has several limitations that should be acknowledged. The current findings are based on transcript-level profiling of 11 PRP genes, and the relationships between changes in PRP gene expression and the observed cell wall structural or membrane integrity phenotypes have not been directly validated at the protein level. Future work should incorporate Western blotting, immunolocalization, and co-expression network analysis to characterize PRP accumulation and spatial distribution. Functional validation through overexpression or CRISPR/Cas9-based knockout of the identified candidate genes—particularly *OsPRP1.1*, *OsPRP3*, *OsRePRP2.1*, *OsHyPRP18*, and *OsHyPRP27*—is essential for determining whether these genes play causal roles in Pro-mediated Cr(VI) tolerance in rice. The results of the present study serve as a foundation for identifying priority targets for downstream functional validation.

## 4. Materials and Methods

### 4.1. Rice Seedlings and Exposure Regimes

Rice seeds (*Oryza sativa* L. XZX 45) were soaked in deionized water for 12 h, evenly spread in plastic cups filled with sandy soil, and cultivated in an artificial climate chamber (light intensity: 20,000 lux; temperature: 25 °C ± 0.5 °C; humidity: 60 ± 2%). The seedlings were irrigated regularly with an appropriate amount of modified 8692 nutrient solution [86]. After 16 days of growth, uniformly developed rice seedlings of similar weight were selected and rinsed with distilled water for subsequent experiments. The treatment protocols for the rice seedlings were as follows:

“Cr(VI)” treatment: Sixteen-day-old rice seedlings were placed in nutrient solution containing different concentrations of potassium chromate [K_2_CrO_4_, Cr(VI)], i.e., 2.0, 8.0, and 16.0 mg/L, with the 0 control group.

“Pro + Cr(VI)” treatment: Sixteen-day-old rice seedlings were first exposed to a nutrient solution containing 1.0 mmol/L Pro for 12 h [87] and then the Pro-treated seedlings were subjected to K_2_CrO_4_ at different concentrations, i.e., 2.0, 8.0, and 16.0 mg/L [88], with the Pro-treated control group.

The Cr concentrations were selected on the basis of previous studies. All the chemical reagents used in this study, including K_2_CrO_4_, were of analytical grade [88]. All flasks were wrapped in aluminum foil to inhibit algal growth and reduce evaporation to a minimum level.

### 4.2. Subcellular Distribution of Total Cr in Rice Plants

After 3 days of exposure, fresh rice seedling tissues (0.5–0.8 g) were homogenized to a slurry in 4 °C grinding medium (containing 50 mM MES-Tris buffer (pH 7.8), 0.25 mM sucrose, 1 mM MgCl_2_, and 10 mM cysteine). The homogenate was then centrifuged at 300× *g* for 30 s, and the pellet (a) was collected as the cell wall fraction. The resulting supernatant (b) was transferred to a new centrifuge tube and centrifuged at 20,000× *g* for 45 min. The subsequent supernatant (c) was collected as the cytoplasmic fraction, and the pellet (d) was designated the organelle fraction. The cell wall (a), cytoplasm (c), and organelle fractions (d) were subsequently separately digested in a digestion mixture of HNO_3_ and HClO_4_ (4:1, *v*/*v*). Finally, the Cr content in the subcellular fractions was analyzed by inductively coupled plasma atomic emission spectrometry (ICP-AES, PerkinElmer Optima 700DV, PerkinElmer, Waltham, MA, USA). The detection limit, determined as the mean of the blank plus 3 times the standard deviation of 10 blanks, was 0.07 μg Cr/L [86].

### 4.3. Determination of Electrolyte Leakage

Electrolyte leakage (EL) from the rice seedlings was measured according to our pre-vious study [54]. Briefly, 0.2 g of fresh rice seedling tissue was accurately weighed, cut into small pieces, and immersed in 5 mL of deionized water. The mixture was then shaken at room temperature for 45 min. The initial conductivity (EC1) of the solution was measured. The sample was subsequently placed in a boiling water bath for 10 min. After cooling, the final conductivity (EC2) was measured. EL was estimated by using the equation (EC1/EC2) × 100%.

### 4.4. Determination of Cell Wall Thickness

Rice root and shoot tissues were cut into 0.5–1.0 cm segments and then fixed in PFA overnight at 4 °C. The following day, the samples were washed three times (5 min each) with 1× PBS, dehydrated in a 30% sucrose solution (prepared with PBS) for 24 h and stored at 4 °C for subsequent experiments. The rice seedling tissues were embedded in Jung OCT embedding medium, and 60 μm thick sections were cut with a Leica cryostat microtome (Leica CM 1858, Leica Camera AG, Wetzlar, Germany). The sections were stained with 1% phloroglucinol solution (prepared in 95% ethanol) for 2 min, and then color-developed with 25% HCl for 2 min. Subsequently, the stained samples were observed and photographed under a microscope. Cell wall thickness was measured using Motic Images Plus software (v. 3.1), with 10 regions selected from each of four independent biological replicates (*n* = 40).

### 4.5. Identification of PRP Genes Involved in Cell Structure Regulation

It was found that plant PRP genes are involved in regulating cell wall synthesis, altering cell wall thickness, maintaining cell membrane integrity, and regulating defense responses (Table 1). Forty-eight isogenes were found in the rice PRP genome family from the China Rice Data Center (https://www.ricedata.cn/gene/index.htm, accessed on 6 February 2024) and the RAP-DB (https://rapdb.dna.naro.go.jp/, accessed on 6 February 2024). The seven functionally characterized genes from Table 1 and the 48 candidate OsPRP genes were used to construct a phylogenetic tree, which was constructed using the maximum likelihood method in MEGA-X software (MEGA 7.0.26), with 500 bootstrap replicates. The tree was subsequently beautified through the iTOL online website (https://itol.embl.de/, accessed on 3 June 2024). Consequently, 12 OsPRPs were identified as candidates on the basis of their close clustering with the seven functionally characterized PRPs with a bootstrap support value exceeding 50% (as shown in Figure 4).

### 4.6. RNA Extraction and RT-qPCR Analysis

The rice seedling tissues, which had been exposed for 3 days (approximately 0.2 g) were rapidly ground into a fine powder in liquid nitrogen. Total RNA was extracted from the samples using a UItrapure RNA Kit (purchased from CWBIO, Kangwei Century Biotechnology Co., Ltd., Beijng, China), and cDNA was synthesized via reverse transcription with a HiFiScript cDNA Synthesis Kit. qRT-PCR was performed using *OsGAPDH1* as the reference gene, and the relative expression levels of target genes were calculated using the 2^−ΔΔCt^ method. Among the 12 candidate genes initially selected, effective primers for specifically amplifying the cDNA of *OsRePRP1.2* could not be designed. Consequently, the remaining 11 genes involved in cell wall structure regulation were successfully analyzed, and their specific primers are listed in Table S1.

### 4.7. Calculation of Gene Expression Variation Factors (GEVFs)

The *GEVFs* (%) for PRPs were calculated by comparing the fold-change in gene expression between the Cr(VI) treatment and the Pro + Cr(VI) treatment in rice [89]. The formula used was as follows:

$$\mathrm{GEVFs}\;=\;\frac{{\mathrm{FC}}_{\left(\mathrm{Pro}+\mathrm{Cr}\right)}-{\mathrm{FC}}_{\left(\mathrm{Cr}\right)}}{{\mathrm{FC}}_{\left(\mathrm{Cr}\right)}}\;\times \;100\%$$

where FC (Pro+Cr) represents the fold change in gene expression in the “Pro + Cr(VI)” treatment, and FC (Cr) represents the fold change in gene expression in the “Cr(VI)” treatment. A *GEVF* threshold of >25% or <−25% was adopted from Zhang et al. [89], in which mathematical modeling of the fold-change between two treatment comparisons established that this threshold corresponds to a statistically meaningful regulatory effect (*p* < 0.05). A positive *GEVF* indicates Pro-mediated gene promotion, whereas a negative *GEVF* indicates repression.

### 4.8. Data Analysis

All of the experimental procedures were independently conducted in quadruplicate (*n* = 4), and the results are expressed as the means ± standard deviations in both the figures and the tables. All experimental data were subsequently subjected to analysis of variance (ANOVA) and Tukey’s multiple range test, with the significance level *α* set at 0.05, enabling comparisons between the treatment groups and the control. The distinct letters accompanying values within individual graphs indicate statistical significance (*p* < 0.05).

## 5. Conclusions

This study investigated the transcriptional responses of PRP genes to Pro_(exo)_ treatment and their association with cell wall structural integrity and membrane stability in rice seedlings under Cr(VI) stress. Our findings demonstrate that Pro_(exo)_ increases Cr sequestration in the root cell walls of rice seedlings, reduces Cr distribution in the shoot cytoplasm and organelles, decreases electrolyte leakage, and increases cell wall thickness. Through qRT-PCR and *GEVFs* analysis of 11 PRP genes involved in cell wall assembly, synthesis, and membrane integrity *OsPRP1.1*, *OsPRP3*, *OsRePRP2.1*, *OsHyPRP18*, and *OsHyPRP27* were identified as the genes most consistently associated with Pro_(exo)_ treatment in roots, with *OsHyPRP27* additionally showing responsiveness in the shoots. These transcriptional associations, combined with the observed changes in cell wall thickness and subcellular Cr distribution, suggest that Pro_(exo)_ may contribute to cellular structural stability under Cr(VI) stress in part through the modulation of these PRP genes. Given that the present study is based on transcript-level profiling and phylogenetic inference without direct protein-level or functional validation, the identified genes should be regarded as priority candidates for future mechanistic investigation. This work provides a transcriptomic foundation for understanding how Pro_(exo)_ may regulate PRP-mediated cell wall function and Cr(VI) tolerance in rice seedlings.

## Figures and Tables

**Table 1:** The functions of PRP genes in plants.

Plant	Gene	Gene Function	Reference
Tobacco (*Nicotiana tabacum*)	*NtHyPRP1*	Involved in cell wall synthesis, promotes cell expansion/elongation	[42]
Cotton (*Gossypium hirsutum*)	*GbHyPRP1*	Negatively regulates resistance to *Verticillium dahliaeby* modulating cell wall thickness and ROS accumulation	[43]
Cotton (*Gossypium hirsutum*)	*GhHyPRP4*	Participates in seedling development under cold stress	[44]
Trifoliate orange (*Poncirus trifoliata* (L.) *Raf.*)	*PtrPRP*	Enhances cold tolerance by maintaining membrane integrity and ROS homeostasis	[45]
Tomato (*Solanum lycopersicum*)	*THyPRP*	Key regulator of flower abscission; silencing inhibits expression of cell wall-modifying enzyme-encoding genes	[46]
Pepper (*Capsicum annuum*)	*CaHyPRP1*	Positive regulator of cell death and negative regulator of basal defense against pathogens	[47]
*Arabidopsis thaliana*	*EARLI1*	Supports seed germination and early development under low-temperature and salt stress	[48]

## Data Availability

Data are contained within the article or Supplementary Materials.

## Supplementary Materials

The following supporting information can be downloaded at: https://www.mdpi.com/article/10.3390/ijms27156669/s1.
