# Supplementary material for: Exogenous Proline Maintains Cell Wall Structure and Membrane Integrity in Rice Seedlings Under Cr(VI) Stress Associated with Regulation of Proline-Rich Proteins
Source: Int J Mol Sci. 2026 Jul 26;27(15):6669. doi: 10.3390/ijms27156669 (PMC13466983; doi:10.3390/ijms27156669)
Supplement: Supplementary file 1 [file ijms-27-06669-s001.zip › ijms-4370125-supplementary.pdf]

Supporting information for

Exogenous proline maintains cell wall structure and membrane integrity in rice seedlings under Cr(VI) stress associated with regulation of proline-rich proteins

Cai-Mei Wang[1], Xue-Qian Wang[1], Ben-Tao Yao [1], Qing Zhang[1, 2], Yu-Juan Lin [1, 2, 3*], Yan-Peng, Liang[1, 2]

1. Guangxi Key Laboratory of Environmental Pollution Control Theory and Technology, Guilin University of Technology, Guilin, 541006, China;
2. University Engineering Research Center of Watershed Protection and Green Development, Guangxi, Guilin University of Technology, Guilin, 541006, China;
3. Collaborative Innovation Center for Water Pollution Control and Water Safety in Karst Area, Guilin University of Technology, Guilin, 541006, China

***Corresponding author**

Prof. & Dr. Yu-Juan Lin, ORCID iD: 0000-0003-3978-4669, E-mail: yujuan@glut.edu.cn

Table S1. PRPs involved in cell wall structure regulation

Gene	Locus identifier	Primersequences (5′-3′)	Ampliconsize (bp)	Accession numbers
OsHyPRP18	LOC_Os06g43600.2	F-GCTCAAGCTCCTCAACATCAAC<br>R-CTAGATGGAGCAGGTGTAGCC	100	XM_015785820.3
OsHyPRP17	LOC_Os06g07220.1	F-GTCTTTCCTGCTCGTCGTC<br>R-AGGTTTGTGGTGGTGGTG	173	XM_015788005.3
OsHyPRP27	LOC_Os10g11370.1	F-CCGTTCTCCTATCGCCAGTC<br>R-GCTGAGCCGTAGAGATATGAGT	103	XM_015758467.3
OsPRP3	LOC_Os10g05750.1	F-TCTTCCACTACAAGAACCCGTC<br>R-GATCAATGGCAGCAGCTTCTTG	254	XM_015759110.3
OsPRP1.1	LOC_Os10g05970.1	F-CTAGTTCTCCTCGGCGTTTG<br>R-GAGCTTTCCAGTGGCCTTG	201	XM_015759083.3
OsPRP1.2	LOC_Os10g05980.1	F-GGCAAGACCAGCTATCCGTC<br>R-GTTCCGACTTTGGCTCAGG	247	XM_015759082.3
OsPRP1.3	LOC_Os10g05990.2	F-GTGAGTACGAGAGCAAGGC<br>R-GCTGATGGGTAGTGTGTCTT	208	XM_015759080.3
OsPRP1.4	LOC_Os10g06000.1	F-GAACGGCAATGGTGAGTACGA<br>R-ACGATCTTGGATGGCTCTTGTC	162	XM_015759081.3
OsRePRP1.1	LOC_Os05g13900.1	F-TCCGATGCCTGAACCTGAACCA<br>R-GTGCTTGTTCGGCGGTGCTAT	133	XM_015782457.3
OsRePRP2.1	LOC_Os07g23660.1	F-AACCTGAGCCGAGTCCTAAC<br>R-TTCCCGGGCACAATTATAGC	151	XM_015789896.3
OsRePRP2.2	LOC_Os07g23640.1	F-TCCTCTCACTGTGCTTCCAT<br>R-GCTTTGTCTCTGGTTGTGGT	162	XM_015789896.3
OsGAPDH	LOC_Os08g03290.1	F-GACAGCAGGTCGAGCATCTTC<br>R-CAGGCGACAAGCTTGACAAAG	74	NM_001403382.1
